# Health Behaviours in Soccer Support Staff: 24-Hour Movement Adherence Is Positively Associated with Diet Quality

**DOI:** 10.3390/sports14060224

**Published:** 2026-05-29

**Authors:** Olivia C. Coope, Tilly J. Spurr, Alex L. Levington, Tom Davies, Beth Lloyd, Enrique Jordán, Blanca Roman-Viñas

**Affiliations:** 1 Blanquerna School of Health Sciences, Ramon Llull University, 08025 Barcelona, Spain; 2 School of Sport, Science and Engineering, University of Chichester, College Lane, Chichester PO19 6PE, UK; m.spurr@chi.ac.uk; 3 Strength and Nutrition Ltd., Brighton BN3 8PS, UK; alex@strengthandnutrition.co.uk; 4 Cheltenham Town Women Football Club, Cheltenham GL52 3PD, UK; 5 Faculty of Social and Behavioural Sciences, Leiden University, 2333 AK Leiden, The Netherlands; 6 Rise Sports Clinic, Carrer de Padilla 285, 08025 Barcelona, Spain; 7 Blanquerna School of Psychology, Education and Sport Sciences, Ramon Llull University, 08022 Barcelona, Spain; blancarv@blanquerna.url.edu

**Keywords:** football staff, multidisciplinary teams, physical activity, sedentary behaviour, sleep, nutrition, lifestyle behaviours, workplace health, cross-sectional study

## Abstract

Soccer support staff operate under demanding schedules and high-performance environments while guiding players’ movement, sleep, and nutrition; however, their own lifestyle behaviours remain under-researched. This exploratory study assessed adherence to the Canadian 24-Hour Movement (24HM) guidelines and its association with diet quality (DQ) in professional and semi-professional soccer support staff. Methods: A cross-sectional survey collected data from 236 staff in the United Kingdom and Spain. Movement behaviours were measured using the Whole Day Matters Toolkit and DQ using the validated Mini-EAT questionnaire. A graded 24HM score (0–8) summed binary adherence across four general (MVPA, LPA, sedentary time, sleep) and four secondary (muscle-strengthening, sedentary interruptions, screen time, sleep–wake time) behaviours. Associations with DQ were estimated using adjusted multiple linear regression. Results: Only 7.6% of participants met all eight guidelines. Each one-point increase in the graded score was associated with 0.89-point higher DQ (95% CI 0.29–1.49, *p* = 0.004), with stronger associations observed for secondary behaviours (β = 1.27, *p* = 0.006) than for general behaviours (β = 0.38, *p* = 0.50). Conclusions: A graded 24HM scoring approach showed a graded association with DQ in soccer staff, with secondary movement behaviours showing a stronger association. All findings should be interpreted as exploratory and hypothesis-generating. ClinicalTrials.gov: NCT06771752.

## 1. Introduction

The demanding nature of professional soccer, including irregular schedules, travel demands, and high-pressure decision-making, places considerable strain on physical and mental well-being. These factors may contribute to fatigue, psychological stress, increased injury risk, and burnout, particularly during congested fixture periods where teams may play multiple matches per week across long competitive seasons [1,2,3,4]. While much of the existing research has focused on understanding and improving the outcomes of these demands on athletes, there is comparatively little literature on multidisciplinary support staff who experience similar schedules. This group, including coaches, medical teams, analysts, nutritionists and other support personnel, faces similar challenges including fatigue, mental health issues, and the pressures of maintaining performance under high-stress conditions [5].

Research into the well-being of team staff within elite sports settings has been limited. A critical gap was identified in recovery-focused strategies for team staff, noting that much of the attention on recovery remains concentrated on athletes [6], highlighting the need for tailored recovery interventions for staff, including educational resources on recovery and the identification of organisational fatigue indicators and barriers that hinder self-care. A study that explored sleep patterns and psychological distress in 78 coaches and high-performance support staff [7] revealed that levels of mental health symptoms in this group were similar to those found in elite athletes. These findings demonstrate that support staff in sports environments face mental and emotional demands that are frequently overlooked, despite their crucial role in athlete well-being.

Support staff in elite sport environments are exposed to long working hours and high-pressure situations [8]. These factors can disrupt physical activity patterns and sleep quality and contribute to the risk of cancer, stroke, and cardiovascular disease (CVD) [9]. Time-pressured environments may also shape dietary behaviours, as staff with limited time may rely on easily accessible online nutrition content rather than formal guidance. A substantial proportion of such content is not produced by qualified healthcare professionals, raising concerns about the accuracy of information consumed by sports staff [10] and motivating the focus on diet quality (DQ) in this study.

The Canadian 24-Hour Movement (24HM) guidelines, which include recommendations on physical activity, sedentary behaviour, and sleep, have been shown to play a significant role in enhancing overall health [11] and are widely used in the scientific literature [12,13,14,15,16,17]. For adults aged 18 to 64 years, the guidelines recommend at least 150 min of moderate-to-vigorous intensity physical activity (MVPA) per week, several hours of light physical activity (LPA), no more than eight hours of sedentary time (ST) per day, and at least seven hours of good-quality sleep per night (SLEEP), with additional recommendations for muscle-strengthening training on two or more days per week (MST), no more than three hours of screen time per day (SCREEN), interrupting ST with movement after one hour (SI) and consistent sleep–wake time (SWT) [18]. The Whole Day Matters Toolkit (WDMT) was developed by The Canadian Society for Exercise Physiology to assist professionals in assessing 24HM by incorporating the guidelines for adults into a set of practical questions [19]. Although developed in Canada, the 24HM guidelines have contributed to an international shift towards integrated movement–behaviour recommendations that consider physical activity, sedentary behaviour, and sleep across the whole 24-h day [18]. The Mini-EAT (Mini Eating Assessment Tool) is a parallel brief instrument used in clinical settings to assess DQ [20], with higher scores reflecting healthier dietary patterns. Support staff in elite sport environments often face irregular schedules and frequent travel, which may negatively affect their movement behaviours and DQ [21]. Diets high in added sugars and other obesogenic foods can negatively affect cognitive function [22] and contribute to CVD [23]; poor dietary habits also contribute to fatigue, poor mental health, and impaired well-being [24].

Most existing 24HM analyses operationalise overall adherence as a binary indicator: a participant either meets all guideline thresholds or does not. In high-performance soccer environments, full adherence to all eight thresholds is rare, which limits statistical power and obscures graded associations between movement behaviours and downstream health outcomes such as DQ. A graded score that counts the number of guidelines met preserves the original guideline structure while distributing participants across a meaningful range of values, which may provide a more sensitive estimation of associations.

This study is exploratory and hypothesis-generating. To the authors’ knowledge, no prior study has examined graded adherence to the 24HM guidelines, or its association with DQ, in soccer support staff. The United Kingdom and Spain were selected because of their prominence in global soccer [25]. We hypothesised that greater 24HM adherence, expressed as a graded 0–8 score, would be positively associated with DQ scores. This study does not aim to show cause and effect, but to explore ways to improve lifestyle behaviours in sport settings for soccer staff.

## 2. Materials and Methods

### 2.1. Study Design and Recruitment

This was an observational, cross-sectional study using an online questionnaire. Participants were recruited as a convenience sample, primarily through searches of LinkedIn and publicly available club websites [26]. LinkedIn and club websites were used as they are practical and cost-effective channels through which professional and semi-professional soccer support staff can be identified with publicly listed roles and affiliations.

Eligible participants were aged 18–64, were currently employed in professional or semi-professional soccer in the United Kingdom or Spain, had a publicly verifiable occupation through LinkedIn or a club website, and provided digital informed consent. Individuals working outside soccer or lacking a verifiable public profile were excluded. The questionnaire was distributed via Google Forms, an established platform for clinical questionnaires [27], and remained open from 15 January to 15 March, coinciding with the soccer mid-season. The questionnaire combined items from the WDMT and the Mini-EAT and was offered in either English or Spanish; the Spanish version was translated by a native Spanish-speaking academic with subject expertise (B.R-V.). A formal forward–backward translation procedure was not conducted; however, the translation was reviewed for linguistic clarity. As the Mini-EAT is composed of food–frequency–style items that translate broadly across populations, no further cultural adaptation was undertaken. Independent variables included sex, country, occupation and work setting (men’s, women’s, or both fields of soccer), self-reported weight, and height. Data collection was, and remains, anonymous. All questions were mandatory, so no submitted surveys were excluded for incompleteness.

### 2.2. Movement–Behaviour Variables and the Graded 24HM Score

The WDMT was used to measure SLEEP in hours per day, MVPA in minutes per week, LPA in hours per day, ST in hours per day, and adherence to MST, SI, SCREEN, and SWT. Continuous physical-activity and sedentary-behaviour items were converted to binary adherence indicators against the published 24HM thresholds: SLEEP ≥ 7 h/day, ST ≤ 8 h/day, MVPA ≥ 150 min/week, LPA > 3 h/day, MST ≥ 2 sessions/week, SI (regular movement after 1 h of ST), SCREEN ≤ 3 h/day, and SWT (consistent sleep–wake schedule) [18,19].

Building on these binary indicators, three graded scores were derived to enable more sensitive inference:

- General score (0–4): sum of binary indicators for SLEEP, ST, MVPA, and LPA;
- Secondary score (0–4): sum of binary indicators for MST, SI, SCREEN, and SWT;
- Total score (0–8): sum of all eight binary indicators (i.e., general + secondary).

The graded scoring assigns equal weight to each of the eight guideline components, reflecting the structural design of the 24HM framework, in which each behaviour is presented as a discrete recommendation, rather than an assumption of equivalent physiological importance. The score should therefore be interpreted as an index of overall adherence breadth rather than a weighted estimate of health impact.

### 2.3. Diet-Quality Score

DQ was assessed using the Mini-EAT, a brief food-frequency screening tool validated against the more comprehensive dietary instrument Healthy Eating Index (HEI) [20]. The Mini-EAT covers fruits, vegetables, legumes, nuts and seeds, seafood, whole grains, low-fat dairy, refined grains, high-fat dairy, and sweets. Each item is scored on a 0–9 ordinal frequency scale, with refined grains, high-fat dairy, and sweets reverse-scored, and item scores were summed to produce an overall DQ score. Although the original validation study reported regression-derived item weights, a reproducible weighted scoring algorithm was not provided. Consistent with the structure of the questionnaire and its intended clinical use, an unweighted summed scoring approach was applied, with scoring direction aligned to the validation study [20]. The Mini-EAT is frequency-based and does not capture portion size or total energy intake; therefore, scores reflect dietary patterns rather than quantitative intake.

### 2.4. Participants

The analysis sample comprised 236 male (*n* = 164; 69.5%) and female (*n* = 72; 30.5%) staff employed in professional or semi-professional soccer across men’s and women’s leagues, in the United Kingdom (n = 122; 51.7%) or Spain (n = 114; 48.3%). Mean age was 32 years (SD = 8.79), mean height of 173.70 cm (SD = 18.12), and mean body mass of 76.29 kg (SD = 15.04). Job titles spanned 14 categories. A numerical total of each job is provided in Supplementary Materials, Section S1. The 14 individual job titles were collapsed into three broader job groups for inferential modelling—Technical (n = 80), Medical (n = 54) and Performance (n = 102)—with the constituent titles and complete participant characteristics listed in Table 1. Per-cell counts for job group and country were: Technical (United Kingdom n = 39, Spain n = 41), Medical (United Kingdom n = 25, Spain n = 29) and Performance (United Kingdom n = 58, Spain n = 44). Two preliminary categories from trial registration were not retained: Referee (excluded as referees do not form part of formal soccer staff or club structures) and Physical Therapist (consolidated under Physiotherapist).

### 2.5. Ethics

This study was approved by the Research Ethics Committee of the School of Health Sciences of Blanquerna Institute, University Ramon Llull (CER-FCSB) on 17 December 2024 (Approval number: 2024-12-02). All procedures performed in this study were in accordance with the ethical standards of the institutional research committee and the Declaration of Helsinki. Digitally signed informed consent was required prior to completion of the questionnaire. The study is registered on ClinicalTrials.gov with the ID NCT06771752.

### 2.6. Statistical Analysis

The recruitment target for this study was informed by the sample sizes commonly reported in comparable cross-sectional studies examining 24HM adherence using regression-based analyses, which have typically included approximately 220–250 participants [28,29]. Although a formal a priori power calculation was not performed, a post hoc sensitivity analysis conducted in G*Power (v3.1.9.6) indicated that with *n* = 236, eight predictors, and α = 0.05, the primary model had 83% power to detect an effect size of *f*^2^ = 0.07 (small-to-medium effect). Descriptive statistics summarised the participant characteristics, the eight binary 24HM behaviours, the three graded scores (general 0–4, secondary 0–4, total 0–8), the three-level total adherence band, and DQ. The DQ score distribution was characterised by mean, SD, median, IQR, range, and skewness. Multiple linear regression was used to estimate the adjusted association between the graded total 24HM score (0–8) and DQ, controlling for sex, country, age (years), job group (Technical/Medical/Performance, with Technical as the reference), and work setting (men’s, women’s or both, with men’s as the reference). A complementary model decomposed the total score into its general (0–4) and secondary (0–4) components, modelled simultaneously. Per-coefficient effects are reported for a clearly defined increment (1-point increase, single-category contrast), and the intercept is included to support reproducibility and reader interpretation.

The primary model was re-estimated replacing the graded total score with: (a) the conventional binary total adherence indicator (8/8 vs. not), (b) the three-level total adherence band (Low 0–2, Moderate 3–5, High 6–8), and (c) a partial-credit version of the score that awards 0/0.5/1 points to MVPA, LPA, ST, and SLEEP based on whether participants fully or partially met each threshold. A further sensitivity analysis replaced the primary DQ outcome with the alternative DQ1 scoring. Model fit was compared using R^2^, adjusted R^2^, and AIC.

Variance inflation factors (VIFs), Shapiro–Wilk tests of residual normality, Breusch–Pagan tests of heteroscedasticity, alongside the visual inspection of diagnostic plots were used to evaluate model assumptions for m1 and m2.

Independent samples Welch’s *t*-tests were used to compare DQ between sex and country categories, with Cohen’s d as the effect size. One-way ANOVAs were used for work setting (3 levels) and the original 14-category job title, with eta-squared (η^2^) as the effect size and Tukey’s HSD for post hoc job-title contrasts. ANOVA degrees of freedom are reported. The analytical plan involved multiple regression coefficients, several *t*-tests, two ANOVAs, and Tukey post hoc contrasts; we therefore explicitly framed the present manuscript as exploratory and hypothesis-generating, did not apply formal correction for multiple comparisons, and treated all isolated significant results as preliminary leads requiring confirmation. Statistical significance was set at *p* < 0.05. The *p*-values are reported consistently to three decimal places, with values smaller than 0.001 reported as *p* < 0.001.

## 3. Results

The questionnaire completion rate was 100%, as all questions were mandatory. The primary regression model explained 17.6% of the variance in DQ (R^2^ = 0.176, adjusted R^2^ = 0.147, AIC = 1566). Multicollinearity diagnostics were acceptable (all VIF < 1.5; GVIF^(1/(2Df)) < 1.11). Visual inspection of residuals and the Shapiro–Wilk and Breusch–Pagan tests indicated no strong evidence against normality or homoscedasticity.

### 3.1. Distribution of 24-Hour Movement Adherence and Diet-Quality Behaviours

**Figure 1:**
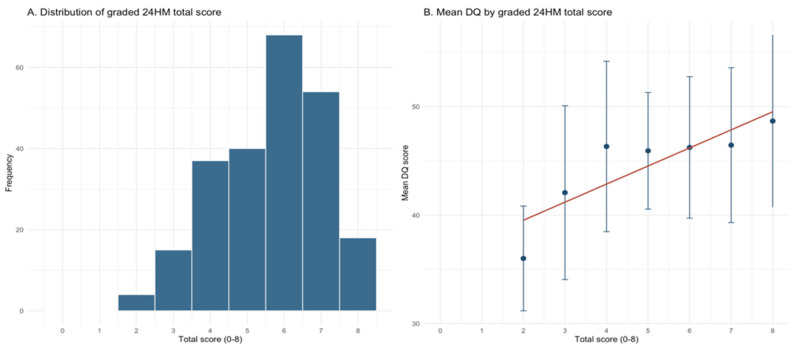
Distribution of graded 24-Hour Movement adherence scores and corresponding diet quality.

Considering the conventional 24HM categories, 36.9% of participants met the General threshold, 14.8% met the Secondary threshold, and only 7.6% met the Total adherence threshold. The graded total score was approximately normally distributed across the 0–8 range, with no participants scoring 0 or 1, four participants (1.7%) scoring 2, and a modal score of 6 (n = 68; 28.8%). The full distribution and corresponding mean Mini-EAT scores are shown in Table 2 and visualised in Figure 1A,B, with the diagonal line representing the linear regression trend.

The DQ score was approximately symmetric and well-dispersed: mean = 46.0, SD = 7.06, median = 46, IQR = 10, range 27–64, skewness = 0.04. The alternative item-level Mini-EAT score (DQ1) had a mean = 39.0, SD = 8.18, median = 40, IQR = 11, range 17–57, and skewness = −0.27. The full Mini-EAT distribution is summarised in Table 2.

In terms of distribution across food categories, 35% of participants consumed 2–3 servings of fruit and 37% consumed 2–3 servings of vegetables per day. Other key nutrient intakes were also low: 27% of participants consumed 1–2 servings of legumes per week, 42% consumed 1–2 servings of fish per week, and 26% consumed 3–4 servings of whole grains per week. Regarding refined grains, 25% of participants consumed 3–4 servings per week. Just under a quarter (23%) of participants consumed 1 serving of low-fat dairy per day, and 28% consumed 1–2 servings of high-fat dairy per week. For sweets, 29% of participants consumed 1–2 servings per week. A breakdown is provided in Supplementary Materials, Section S2.

### 3.2. Adjusted Association Between Graded 24HM Adherence and Diet Quality

**Figure 2:**
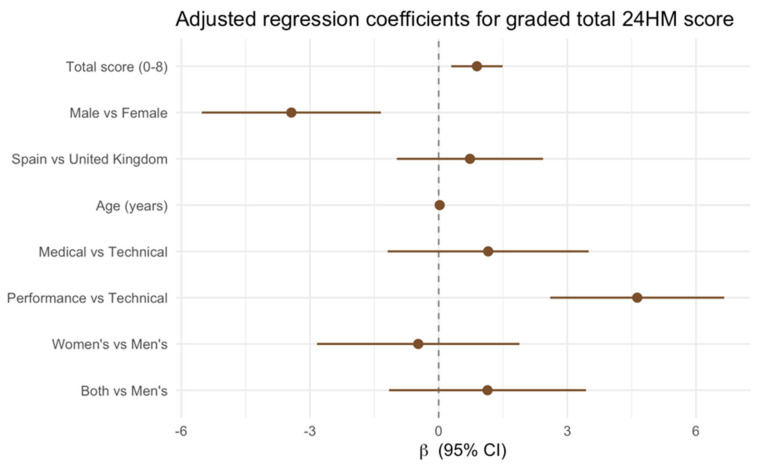
Adjusted regression coefficients for diet quality on graded 24-Hour Movement total score.

In the primary adjusted regression model, the graded 24HM total score was positively associated with DQ. Each one-point increase in the total score (a 1-unit increase on the 0–8 scale) was associated with a 0.892-point higher DQ score (95% CI 0.293 to 1.491, *p* = 0.004), corresponding to an expected 7.14-point difference across the full 0–8 range. Male staff had a lower adjusted Mini-EAT score than female staff (β = −3.43, 95% CI −5.52 to −1.35, *p* = 0.001). Performance roles had a higher adjusted Mini-EAT score than the reference Technical group (β = 4.63, 95% CI 2.60 to 6.66, *p* < 0.001), whereas Medical roles did not differ significantly from Technical (β = 1.15, *p* = 0.333). Country (Spain vs. the United Kingdom), age, and work setting were not significantly associated with DQ in the adjusted model. Coefficients are presented in Table 3 and visualised in Figure 2.

To clarify which side of the 24HM guideline structure was driving the overall association, the model simultaneously included the general (0–4) and secondary (0–4) scores. The secondary score remained associated with DQ (β = 1.27, 95% CI 0.37 to 2.16, *p* = 0.006), whereas the general score did not (β = 0.38, 95% CI −0.72 to 1.47, *p* = 0.500). This pattern suggests that the positive graded association between 24HM adherence and DQ is more strongly related to the secondary group (MST, SI, SCREEN, SWT) than to the four general behaviours (MVPA, LPA, ST, SLEEP). All results should be interpreted as exploratory descriptions of where the overall association sits.

### 3.3. Sensitivity and Robustness Analyses

Model fit statistics are reported in Table 4. The graded total score (adjusted R^2^ = 0.147), the three-level band (adjusted R^2^ = 0.157), and the partial-credit version (adjusted R^2^ = 0.150) all outperformed the conventional binary indicator (adjusted R^2^ = 0.125), and the binary indicator was non-significant in the adjusted model. This indicates that prior null findings using binary 24HM adherence may reflect loss of statistical power from the coarse categorisation rather than a true absence of association. The DQ1 scoring produced a similar coefficient direction for total score but with a higher AIC (1641).

### 3.4. Exploratory Group Comparisons

**Figure 3:**
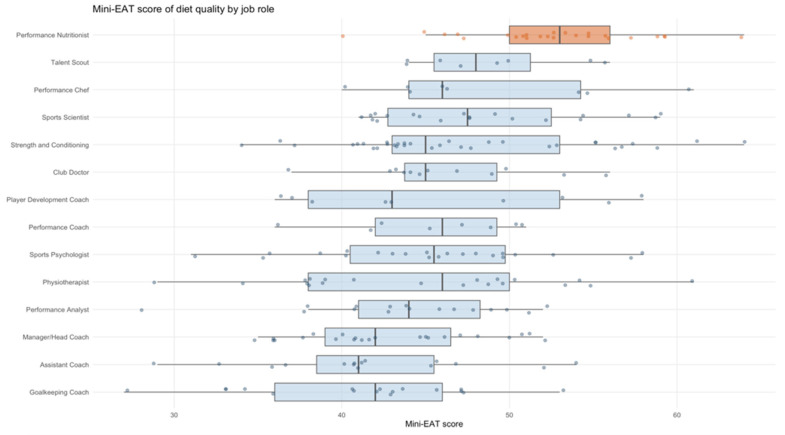
Diet quality scores of soccer staff by job title.

Exploratory unadjusted group comparisons (Table 5) used Welch’s *t*-tests for sex and country and one-way ANOVA for work setting and the original 14-category job title, with Cohen’s d and η^2^ reported respectively as effect sizes and Tukey’s HSD used for post hoc job-title contrasts. Female staff reported higher unadjusted Mini-EAT DQ than male staff (small-to-moderate effect, d ≈ 0.43, *p* = 0.002). No unadjusted differences were detected across country (d ≈ 0.11, *p* = 0.387) or work setting (η^2^ ≈ 0.008, *p* = 0.386). Job title showed a significant unadjusted overall effect (η^2^ ≈ 0.21, *p* < 0.001). Tukey post hoc comparisons indicated that Performance Nutritionists had a higher DQ than Assistant Coaches, Goalkeeping Coaches and Manager/Head Coaches (*p* < 0.001), Performance Analysts (*p* = 0.003), Physiotherapists (*p* = 0.006), and Sports Psychologists (*p* = 0.009). Figure 3 shows the DQ score distribution across the 14 job titles, with the highest DQ score shown in orange.

## 4. Discussion

### 4.1. Main Findings

In this exploratory cross-sectional study of multidisciplinary soccer support staff in the United Kingdom and Spain, three main findings emerged. First, when 24HM adherence was operationalised as a graded 0–8 score rather than a binary indicator, a positive association with DQ was evident: each additional guideline met was associated with a 0.89-point higher DQ score, accumulating to a 7.14-point difference between minimum and maximum adherence on the Mini-EAT scale across the full theoretical 0–8 range. Second, decomposing the total score indicated that the 24HM-DQ association was more strongly associated with the secondary group (MST, SI, SCREEN, SWT) than with the general group (MVPA, LPA, ST, SLEEP). Third, differences in DQ were observed across professional roles, with staff in performance-oriented positions demonstrating higher DQ scores than other job groups, and Performance Nutritionists exhibiting the highest scores overall. All findings should be interpreted as preliminary associations from an exploratory analysis.

### 4.2. Comparison with the Existing Literature

Adherence to the 24HM guidelines was low when operationalised in binary terms: 36.9% of participants met the General threshold, 14.8% met the Secondary threshold, and only 7.6% met all eight criteria. These figures broadly align with prior reports of low binary adherence in general populations, including 1.6% of 2338 Latin American adults aged 18–65 years [30], 1.8% of 4562 older Chinese adults [31], and 25.5% of 7059 Chinese adults aged 20–79 years [32]. The graded distribution observed here, however, demonstrates that the apparent floor effect of binary adherence is largely an artefact of summing eight stringent thresholds: most participants met the majority of thresholds (median = 6 of 8). Soccer support staff in this cohort therefore showed a clear gradient of adherence rather than uniform inactivity, and this gradient tracked measurably with DQ.

DQ scores in this cohort suggest that consumption frequencies for fruits, vegetables, legumes, whole grains, and fish were consistently low, in line with the previous literature on suboptimal dietary habits in sporting support staff due to time constraints, irregular schedules, and limited access to tailored nutrition support [21]. While DQ here was moderate, comparable patterns have been observed in broader populations: in a sample of 20,745 Spanish adults, only 2.8% reported consuming vegetables at least three times per day [33], and most adults in England do not meet the recommended intake of five portions of fruit and vegetables per day [34]. A study of 45,459 adults in China found that 79.9% were at risk of inadequate fruit intake and 53% of inadequate vegetable intake against the national dietary guidelines [35]. The moderate DQ observed in soccer support staff therefore appears consistent with broader international trends of suboptimal fruit and vegetable intake in adult populations.

Decomposing the graded total score indicated that the secondary group (MST, SI, SCREEN, SWT) showed a stronger association with DQ in the decomposed model (β = 1.27, *p* = 0.006), whereas the general group (MVPA, LPA, ST, SLEEP) was not independently associated with DQ after adjustment (β = 0.38, *p* = 0.500). The secondary behaviours of structured exercise sessions, regular movement breaks, screen-time limits, and consistent sleep–wake timing may lend themselves more readily to routine-based scheduling than the general behaviours, which are often shaped by broader occupational demands. One plausible interpretation is habit formation theory, which proposes that repeated behaviours in stable contexts become increasingly automatic through cue–response associations [36]. However, the present study did not directly assess habit strength, cue-dependent behaviour, or automaticity, and this interpretation should therefore be considered speculative. Alternative explanations include the possibility that these secondary behaviours are more tightly structured within daily routines, are more sensitive to occupational constraints, or reflect clustering with other health-related behaviours such as dietary practices.

Sex differences were observed: female staff reported higher unadjusted DQ than male staff (d ≈ 0.43, *p* = 0.002), aligning with the literature reporting higher HEI scores in women [37]. The country effect was small and differed between adjusted and unadjusted analyses: country was not significantly associated with DQ in either the adjusted (Spain vs. the United Kingdom β = 0.73, *p* = 0.400) or unadjusted (d ≈ 0.11, *p* = 0.387) comparison. Although the prior literature suggests greater Mediterranean-diet adherence in Spain compared with the United Kingdom [38], the present cohort did not reproduce that pattern, possibly because professional soccer environments share schedule-related constraints across both countries [21].

In this study, 22% of participants reported sleeping fewer than 7 h per night and 44% did not maintain regular sleep–wake time, consistent with previous research in elite sports in which 18.2% of coaches reported moderate sleep disturbances using the ASSQ [7]. Although the WDMT does not directly assess sleep quality, the combination of short sleep duration and irregular sleep timing observed here suggests similar patterns of disrupted sleep behaviours. While sleep duration alone did not emerge as an independent predictor in the per-component decomposition, the SWT indicator forms part of the secondary score that did predict DQ, consistent with the literature linking sleep regularity, appetite regulation, and overall DQ [39].

### 4.3. Implications for Practice

The findings highlight a potential need for soccer organisations to embed staff health into their performance models. Clubs may wish to take a systems-level approach that integrates staff well-being into organisational planning, including structured physical-activity and nutrition policies that mirror the support already provided to players: access to nutritious meals during travel and at training facilities, designated time and space for physical activity, and workload management strategies that reduce ST. Scheduling reforms, such as protecting mealtimes and incorporating micro-breaks, may help alleviate time pressures that restrict healthier choices. Internal education and staff development programmes could include behaviour-change strategies and self-care practices, which may lead to increased physical activity and reduced sedentary behaviour [40]. The results, taken together, suggest that programmes targeting the secondary movement group of muscle-strengthening, scheduled movement breaks, screen-time limits and consistent sleep–wake time, may represent a practical behavioural cluster, which offers a starting point for time-constrained organisations. Performance departments could also track staff well-being using validated, cost-effective tools such as the WDMT and Mini-EAT used here, alongside Total Quality Recovery [41] and the Hamilton Anxiety Rating Scale [42].

Across exploratory unadjusted comparisons, participants working in nutrition roles reported the highest DQ scores. We frame this observation as exploratory and hypothesis-generating: it could reflect greater nutrition literacy and ability to critically appraise online nutrition content [10,43], but it could also reflect self-selection into the survey. Performance Nutritionists in soccer are employed primarily to support players [44], with limited emphasis on the nutritional well-being of staff. Qualified Performance Nutritionists would be well placed to lead broader staff-facing health-promotion initiatives, including education workshops and personalised consultations. Quantitative work has noted that players and stakeholders regard the daily soccer schedule as “too busy to eat” [45], suggesting that structural changes such as protected meal breaks and improved food availability at training venues may be required to allow staff to improve their workplace diet.

### 4.4. Limitations

Several limitations apply. This is an observational, cross-sectional study, so causal relationships cannot be inferred, and the findings represent associations at a single time point. The recruitment strategy is a convenience sample identified through LinkedIn and club websites, which is unlikely to be representative of all soccer support staff in the United Kingdom and Spain, and may over-represent individuals with greater health awareness or engagement with online platforms; the present results should not be generalised beyond the recruited cohort. Accordingly, cross-country comparisons should not be interpreted as psychometrically equivalent. Although the WDMT and Mini-EAT are validated instruments in their original language, the Spanish version was translated for this study without a formal forward–backward translation procedure, which may affect linguistic equivalence and item interpretation among Spanish-speaking participants—this is a key reason for interpreting the country contrast cautiously. Both 24HM and Mini-EAT data are self-reported and may be affected by recall and social-desirability bias, particularly for items that are difficult to consume in high quantities (e.g., multiple daily servings of legumes); the Mini-EAT does not accommodate individual dietary needs such as allergies, intolerances, or cultural variation, which may further limit DQ classification. The analytical plan involved multiple regression coefficients, *t*-tests, ANOVAs, and Tukey post hoc tests; in line with our exploratory framing, we did not apply formal multiplicity correction, and isolated significant findings should be treated as preliminary leads. The graded score is more sensitive than the binary indicator and treats each guideline as equally weighted; future work could explore data-driven weights or item-response-theory scoring. The Mini-EAT scoring approach, although aligned with the original validation study’s direction, is unweighted; the accompanying DQ1 sensitivity analysis is intended to characterise the robustness of the conclusions to scoring choices. While aligned with the original instrument structure, the Mini-EAT scoring in this study does not account for potential differential contributions of individual dietary components to overall DQ. Finally, data were collected during the mid-season, and the cohort spans a wide range of competitive environments (from The FA, La Liga, the Premier League, the FA Women’s Super League, and Liga F to lower-tier and academy-level staff), introducing variability in resources and infrastructure; in addition, the findings may not generalise to off-season or pre-season periods.

### 4.5. Strengths

This study has several strengths. The sample (n = 236) is relatively large for this under-studied population and was recruited internationally across two leading soccer nations. The graded 24HM score introduced here distributes participants meaningfully across the 0–8 range and uncovered an association with DQ that the binary indicator may not detect, with sensitivity analyses yielding consistent directional findings. Reporting included both adjusted and unadjusted analyses with effect sizes (Cohen’s d, η^2^) and ANOVA degrees of freedom, supporting transparent interpretation of the inferential burden. Diagnostic checks supported the assumptions of the linear regression. To the authors’ knowledge, this is the first study to examine graded 24HM adherence in relation to DQ specifically in multidisciplinary soccer support staff.

### 4.6. Future Directions

Future research should examine these relationships in longitudinal and intervention-based designs, ideally tracking staff across the competitive season to capture mid-, off-, and pre-season periods. Targeted workplace interventions on the secondary movement group (MST, SI, SCREEN and SWT) would offer a tractable test of whether changes in 24HM adherence translate into measurable DQ change at the cohort level. A formal forward–backward validation of the Spanish-language WDMT and Mini-EAT would strengthen any future cross-country comparison. Replication in larger samples that include adequately powered subgroups for each individual job title would help determine whether the Performance Nutritionist signal observed here represents a genuine effect or self-selection.

## 5. Conclusions

This exploratory study examined the relationship between adherence to the 24HM guidelines and DQ in multidisciplinary soccer support staff. Adherence to the 24HM guidelines was moderate to low, with only a small proportion of participants (7.6%) meeting all eight movement recommendations, and the DQ scores indicated low consumption frequencies for fruits, vegetables, legumes, whole grains, and fish. Staff working in nutrition roles reported higher DQ scores than several other professions. When 24HM adherence was operationalised as a graded 0–8 score, a positive association with DQ emerged and the secondary movement group (muscle-strengthening, movement breaks, screen-time limits, and consistent sleep–wake time) showed a stronger association with DQ. Demographic and occupational factors, such as female staff and Performance job group, were also associated with DQ. These findings are exploratory and should not be over-interpreted as a categorical mismatch between professional expertise and personal behaviours; they do, however, raise the practical question of whether the lifestyle support extended to players is also being extended to those advising them. The results underline the importance of integrating staff well-being into performance models and advocate for structured health interventions and nutrition education strategies to improve staff health within elite soccer environments. Further research is needed to confirm these relationships and to develop effective health-promotion strategies for soccer support staff, who represent a critical yet underexamined component of the high-performance environment.

## Figures and Tables

**Table 1:** Participant characteristics and job-group structure.

Variable	Category/Constituent Titles	n (Mean ± SD)	%
Total participants	-	236	-
Sex	Male	164	69.5
	Female	72	30.5
Country	United Kingdom	122	51.7
	Spain	114	48.3
Age (years)	-	32 ± 8.79	-
Height (cm)	-	173.70 ± 18.12	-
Body mass (kg)	-	76.29 ± 15.04	-
Work setting	Men’s soccer	130	55.1
	Women’s soccer	59	25.0
	Both	47	19.9
Job group: Technical	Manager/Head Coach, Assistant Coach, Goalkeeping Coach, Performance Coach, Player Development Coach, Talent Scout	80	33.9
Job group: Medical	Club Doctor, Physiotherapist, Sports Psychologist	54	22.9
Job group: Performance	Performance Analyst, Performance Chef, Performance Nutritionist, Sports Scientist, Strength and Conditioning Coach	102	43.2

Notes: Job groups follow the revised three-level structure used in primary regression models. Work setting refers to which fields of soccer the staff member supports.

**Table 2:** 24-Hour Movement adherence distributions and mean diet quality by scoring method.

Variable	Category	n	%	Mean Mini-EAT (SD)
Binary categories (descriptive only)				
General 24HM (4/4)	Not meeting	149	63.1	-
	Meeting	87	36.9	-
Secondary 24HM (4/4)	Not meeting	201	85.2	-
	Meeting	35	14.8	-
Total 24HM (8/8)	Not meeting	218	92.4	-
	Meeting	18	7.6	-
Graded total score (0–8)—primary inferential exposure				
	2	4	1.7	36.0 (4.83)
	3	15	6.4	42.1 (8.01)
	4	37	15.7	46.3 (7.85)
	5	40	16.9	45.9 (5.38)
	6	68	28.8	46.2 (6.52)
	7	54	22.9	46.4 (7.14)
	8	18	7.6	48.7 (7.94)
Three-level total adherence band (corrected)				
	Low (0–2)	4	1.7	36.0 (4.83)
	Moderate (3–5)	92	39.0	45.5 (7.00)
	High (6–8)	140	59.3	46.6 (6.95)
Graded general score (0–4)				
	0	1	0.4	36.0 (-)
	1	5	2.1	47.6 (9.53)
	2	46	19.5	45.4 (8.30)
	3	97	41.1	45.6 (5.86)
	4	87	36.9	46.8 (7.43)
Graded secondary score (0–4)				
	0	7	3.0	39.0 (5.45)
	1	30	12.7	43.9 (7.57)
	2	67	28.4	46.0 (6.65)
	3	97	41.1	46.6 (7.03)
	4	35	14.8	47.5 (6.92)
Mini-EAT outcome distribution				
DQ (primary)	-	236	-	46.0 (7.06); median 46; IQR 10; range 27–64; skewness 0.04
DQ1 (sensitivity)	-	236	-	39.0 (8.18); median 40; IQR 11; range 17–57; skewness −0.27

Notes: Binary categories are reported for description only; the graded total score is the primary inferential exposure. Total band cut-offs were corrected from a previous version to Low 0–2/Moderate 3–5/High 6–8.

**Table 3:** Adjusted multiple linear regression—diet quality score on graded 24-Hour Movement score.

Variable (Unit/Contrast)		β (Mini-EAT Pts)	95% CI	t	*p*-Value
(Intercept) (Mini-EAT pts at reference categories, age = 0)		39.9	34.6 to 45.2	14.8	<0.001 *
Total score (per 1-point increase, 0–8 scale)		0.892	0.293 to 1.491	2.93	0.004 *
Sex: Male vs. Female (reference)		−3.43	−5.52 to −1.35	−3.24	0.001 *
Country: Spain vs. United Kingdom (reference)		0.730	−0.976 to 2.44	0.84	0.400
Age (per 1 year)		0.024	−0.075 to 0.123	0.48	0.635
Job group: Medical vs. Technical (reference)		1.15	−1.19 to 3.50	0.97	0.333
Job group: Performance vs. Technical (reference)		4.63	2.60 to 6.66	4.50	<0.001 *
Work setting: Women’s vs. Men’s (reference)	−0.476	−2.84 to 1.88		−0.40	0.692
Work setting: Both vs. Men’s (reference)	1.14	−1.16 to 3.44		0.98	0.329

Notes: *n* = 236. R^2^ = 0.176, adjusted R^2^ = 0.147, AIC = 1566. All VIFs < 1.5. * *p* < 0.05. CI = confidence interval. Coefficients are expressed in Mini-EAT points; for total score, the coefficient corresponds to a 1-point increase on the 0–8 graded score (i.e., one additional 24HM guideline met). Single-category contrasts use the listed reference category.

**Table 4:** Model fit comparison across alternative 24-Hour Movement adherence operationalisations and Mini-EAT outcomes.

Model	Exposure/Outcome	R^2^	Adj. R^2^	AIC	
m1 (primary)	Graded total score (0–8) = Mini-EAT (DQ)	0.176	0.147	1566	
m2	General + Secondary (0–4 each) = Mini-EAT (DQ)		0.181	0.148	1566
Sensitivity 1	Binary total adherence (8/8 vs. not) = Mini-EAT	0.155	0.125	1572	
Sensitivity 2	Three-level total band (Low/Mod/High) = Mini-EAT	0.190	0.157	1564	
Sensitivity 3	Partial-credit total score (0/0.5/1 weighting) = Mini-EAT	0.179	0.150	1565	
Sensitivity 4	Graded total score = DQ1 (alt. Mini-EAT scoring)	0.157	0.127	1641	

Notes: All models adjusted for sex, country, age, job group (Technical/Medical/Performance), and work setting (men’s, women’s, both); *n* = 236. Lower AIC indicates better fit.

**Table 5:** Exploratory unadjusted group comparisons of the Mini-EAT diet quality.

Comparison	Group	DQ Mean (SD)	Test (df)	Effect Size	*p*-Value
Sex (Welch’s *t*-test)	Female	48.08 (6.70)	t(~138) = 3.07	d = 0.43	0.002 *
	Male	45.07 (7.04)			
Country (Welch’s *t*-test)	United Kingdom	45.61 (7.24)	t(~234) = 0.87	d = 0.11	0.387
	Spain	46.40 (6.87)			
Work setting (one-way ANOVA)	Men’s	45.62 (7.17)	F(2, 233) = 0.96	η^2^ = 0.008	0.386
	Women’s	45.81 (6.69)			
	Both	47.26 (7.21)			
Job title (one-way ANOVA)	14 categories	-	F(13, 222) = 4.42	η^2^ ≈ 0.21	<0.001*

Notes: All tests are unadjusted and exploratory; effect sizes are Cohen’s d (*t*-tests) and η^2^ (ANOVA). *p*-values are reported to three decimal places, with values smaller than 0.001 reported as *p* < 0.001. Tukey post hoc contrasts for the 14-category job-title ANOVA are described in the text. * *p* < 0.05.

## Data Availability

Data supporting the findings of this study are available from the corresponding author upon reasonable request.

## Supplementary Materials

The following supporting information can be downloaded at: https://www.mdpi.com/article/10.3390/sports14060224/s1, Section S1: Staff Roles; Section S2: Distribution of Participants by Frequency of Consumption of Food from the Mini-EAT Questionnaire.

## Abbreviations

24HM—24-Hour Movement; AIC—Akaike information criterion; ASSQ—Athlete Sleep Screening Questionnaire; CI—confidence interval; CVD—cardiovascular disease; DQ—diet quality; General 24HM—MVPA, LPA, ST, and SLEEP; HAM-A—Hamilton Anxiety Rating Scale; HEI—Healthy Eating Index; IQR—interquartile range; LPA—light physical activity; Mini-EAT—Mini Eating Assessment Tool; MST—muscle-strengthening training; MVPA—moderate-to-vigorous physical activity; SCREEN—screen time; Secondary 24HM—MST, SI, SCREEN, and SWT; SD—standard deviation; SI—sedentary interruptions; SLEEP—sleep; ST—sedentary time; DQ1—Alternative Mini-EAT Total Score; SWT—sleep–wake time; Total 24HM—combination of general and secondary 24HM; TQR—total quality recovery; VIF—variance inflation factor; WDMT—Whole Day Matters Toolkit.
