# Supplementary material for: Health Behaviours in Soccer Support Staff: 24-Hour Movement Adherence Is Positively Associated with Diet Quality
Source: Sports (Basel). 2026 May 29;14(6):224. doi: 10.3390/sports14060224 (PMC13306739; doi:10.3390/sports14060224)
Supplement: Supplementary file 1 [file sports-14-00224-s001.zip › Supplementary Material.pdf]

Supplementary Material

S1. Staff roles

The roles included Manager/Head Coach (n = 23), Assistant Coach (n = 15), Club Doctor (n = 12), Goalkeeping Coach (n = 17), Performance Analyst (n = 16), Performance Chef (n = 8), Performance Coach (n = 8), Performance Nutritionist (n = 25), Physiotherapist (n = 20), Player Development Coach (n = 9), Sports Psychologist (n = 22), Sports Scientist (n = 20), Strength and Conditioning (n = 33), and Talent Scout (n = 8).

S2. Distribution of Participants by Frequency of Consumption of Food from the Mini-EAT Questionnaire

Distribution of Participants by Frequency of Consumption of Food from the Mini-EAT Questionnaire

**Variables**		**n**	**%**
**Fruit intake**			
	No consumption	6	2.54
	Less than one serving per week	11	4.66
	One to two servings per week	20	8.47
	Three to four servings per week	35	14.83
	Five to six servings per week	12	5.08
	One serving per day	37	15.68
	Two to three servings per day	83	35.17
	Four to five servings per day	26	11.02
	Six or more servings per day	6	2.54
**Vegetable intake**			
	No consumption	3	1.27
	Less than one serving per week	5	2.12
	One to two servings per week	13	5.51
	Three to four servings per week	29	12.29
	Five to six servings per week	22	9.32
	One serving per day	31	13.14
	Two to three servings per day	87	36.86
	Four to five servings per day	35	14.83
	Six or more servings per day	11	4.66
**Legumes intake**			
	No consumption	15	6.36
	Less than one serving per week	19	8.05
	One to two servings per week	64	27.12
	Three to four servings per week	53	22.46
	Five to six servings per week	14	5.93
	One serving per day	41	17.37
	Two to three servings per day	28	11.86
	Four to five servings per day	2	0.85

	Six or more servings per day	0	0
**Fish intake**			
	No consumption	26	11.02
	Less than one serving per week	32	13.56
	One to two servings per week	99	41.95
	Three to four servings per week	64	27.12
	Five to six servings per week	7	2.97
	One serving per day	5	2.12
	Two to three servings per day	3	1.27
	Four to five servings per day	0	0
	Six or more servings per day	0	0
**Wholegrains intake**			
	No consumption	25	10.59
	Less than one serving per week	26	11.02
	One to two servings per week	34	14.41
	Three to four servings per week	61	25.85
	Five to six servings per week	15	6.36
	One serving per day	38	16.1
	Two to three servings per day	34	14.41
	Four to five servings per day	3	1.27
	Six or more servings per day	0	0
**Refined grains intake**			
	No consumption	26	11.02
	Less than one serving per week	18	7.63
	One to two servings per week	48	20.34
	Three to four servings per week	58	24.58
	Five to six servings per week	24	10.17
	One serving per day	39	16.53
	Two to three servings per day	22	9.32
	Four to five servings per day	0	0
	Six or more servings per day	1	0.42
**Low-fat dairy intake**			
	No consumption	34	14.41
	Less than one serving per week	18	7.63
	One to two servings per week	36	15.25
	Three to four servings per week	45	19.07
	Five to six servings per week	22	9.32
	One serving per day	54	22.88
	Two to three servings per day	24	10.17

	Four to five servings per day	2	0.85
	Six or more servings per day	1	0.42
**High-fat dairy and saturated fat intake**			
	No consumption	19	8.05
	Less than one serving per week	34	14.41
	One to two servings per week	65	27.54
	Three to four servings per week	43	18.22
	Five to six servings per week	10	4.24
	One serving per day	44	18.64
	Two to three servings per day	18	7.63
	Four to five servings per day	2	0.85
	Six or more servings per day	1	0.42
**Sweets and sweet foods intake**			
	No consumption	20	8.47
	Less than one serving per week	39	16.53
	One to two servings per week	68	28.81
	Three to four servings per week	53	22.46
	Five to six servings per week	14	5.93
	One serving per day	34	14.41
	Two to three servings per day	8	3.39
	Four to five servings per day	0	0
	Six or more servings per day	0	0

n = number of participants; % = percentage; Mini-EAT = Mini-Eating Assessment Tool; DQ = diet quality.
